# The mediating role of cognitive flexibility in the relationship between social support and non-suicidal self-injury among adolescents: a multicenter cross-sectional study

**DOI:** 10.3389/fpsyg.2025.1546751

**Published:** 2025-03-14

**Authors:** Chenchen Wang, Jiaqi Zheng, Guanghui Shen, Hong Chen, Xinwu Ye, Cheng-Han Li, Bin Wu

**Affiliations:** ^1^Department of Psychiatry, Wenzhou Seventh People's Hospital, Wenzhou, China; ^2^School of Mental Health, Wenzhou Medical University, Wenzhou, China; ^3^Deparment of Mental Health, The First Affiliated Hospital of Wenzhou Medical University, Wenzhou, China; ^4^Third Affiliated Hospital of Wenzhou Medical University, Huzhou, China; ^5^Huzhou Third Municipal Hospital, Huzhou, China

**Keywords:** adolescents, cognitive flexibility, neuropsychological, non-suicidal self-injury, social support

## Abstract

**Background:**

Non-suicidal self-injury (NSSI) is a prevalent and concerning behavior among adolescents worldwide, particularly in China. This study aimed to investigate the relationships between social support, cognitive flexibility, and NSSI, and to examine the potential mediating role of cognitive flexibility in the association between social support and NSSI among adolescents.

**Method:**

A multicenter cross-sectional study was conducted with 2,109 adolescents (aged 12–18 years, mean age 15.00 ± 1.65 years, 52.3% female) recruited from 14 psychiatric hospitals/outpatient clinics. Participants completed self-report measures of social support and NSSI, along with a cognitive flexibility assessment using the Wisconsin Card Sorting Test. Structural equation modeling was employed to test the hypothesized mediation model.

**Results:**

Social support was negatively associated with NSSI frequency (*β* = −0.11, *p* < 0.001) and positively related to cognitive flexibility (*β* = 0.09, *p* < 0.001). The mediation effect of cognitive flexibility was statistically significant [*β* = −0.01, 95% CI (−0.01, −0.01)] accounting for 8.33% of the total effect of social support on NSSI. Multi-group analysis revealed that the association between social support and cognitive flexibility was stronger in females (*β* = 0.11, *p* < 0.001) compared to males (*β* = 0.02, *p* > 0.05).

**Conclusion:**

This multicenter study provides evidence for the protective roles of social support and cognitive flexibility against NSSI in adolescents. The findings suggest that cognitive flexibility partially mediates the relationship between social support and NSSI, with notable gender differences. These results highlight the importance of enhancing both social support and cognitive flexibility in interventions aimed at reducing NSSI among adolescents.

## 1 Introduction

Non-suicidal self-injury (NSSI) is increasingly recognized as a prevalent and worrisome behavior among adolescents. Defined as deliberate self-harm without suicidal intent, NSSI is often used as a coping mechanism for emotional distress and has varying prevalence rates among adolescents. Studies have shown lifetime prevalence rates of 7.5 to 8% in preadolescents, increasing to 12 to 23% in adolescents (Washburn et al., 2012). A comprehensive meta-analysis covering data from 1989 to 2018 reported lifetime and 12-month prevalence rates of NSSI of 22.1 and 19.5% for children and adolescents, respectively (Lim et al., 2019). NSSI typically begins in early to mid-adolescence, usually peaks around the age of 14–15 years, and tends to decrease in late adolescence.

Adolescents with NSSI behaviors are at higher risk for adverse outcomes, including mental health disorders and an increased likelihood of future suicidal behaviors. The high prevalence of NSSI across populations, including a lifetime prevalence of 27.6% among European adolescents underscores the need to understand the underlying causes and associated factors (Brunner et al., 2013). This understanding is essential for developing targeted prevention and intervention strategies.

### 1.1 Cognitive flexibility in adolescents

Cognitive flexibility is an important component of adolescents’ cognitive development and represents the mental ability to shift between concepts and to think about multiple concepts simultaneously. This ability is essential for adapting to new and unexpected situations, problem solving and decision making. Cognitive flexibility plays a pivotal role in adolescent mental health. It enables adolescents to better cope with the emotional and social complexities of this developmental stage.

Research suggests that cognitive flexibility can moderate the relationship between maltreatment and emotion regulation in adolescents with childhood trauma (Bozorgi Kazerooni and Gholamipour, 2023). Additionally, among overweight adolescents, cognitive inflexibility is associated with elevated emotion-driven impulsivity, suggesting a potential relationship between cognitive flexibility and impulsivity (Martín-Rodríguez et al., 2022). A growing body of research suggests that higher levels of cognitive flexibility are associated with greater emotion regulation, lower impulsivity, and lower propensity to engage in risky behaviors, including NSSI (Chen et al., 2023). Cognitive flexibility allows adolescents to find alternative coping strategies in stressful situations, which reduces the likelihood of resorting to undesirable behaviors (e.g., NSSI). Adolescents’ engagement in risk-taking and novelty-seeking behaviors is also influenced by their cognitive flexibility, as these behaviors require the ability to adapt to changing environments and assess potential consequences (Ciranka and van den Bos, 2021).

Moreover, cognitive flexibility in adolescence is closely tied to the development of adaptive decision-making skills, particularly in the context of reward prediction error processing, highlighting its relevance in learning from experiences and adjusting behavior accordingly (Heffner et al., 2021).

### 1.2 Role of social support

Social support is another key factor influencing adolescent mental health (Wang et al., 2021). It encompasses the perceived and actual support that individuals receive from their social networks, including family, friends and other important relationships. Research has consistently shown that a strong social support system has a protective effect on adolescent mental health and can provide a buffer against a variety of psychological problems. In particular, research has shown that adolescents with strong social support networks are less likely to engage in self-harming behaviors, such as non-self-harming behaviors.

For example, one study found that social support was a partial mediator between childhood disadvantage and NSSI, suggesting that adolescents with lower social support were more likely to engage in NSSI (Zhou et al., 2024). Another study emphasized that ACE (Adverse Childhood Experiences) and low social support were associated with increased risk of NSSI and suicide among Chinese adolescents (Fan et al., 2021). In addition, an Iranian study showed that weak family psychological functioning and low levels of perceived social support significantly increased the likelihood of NSSI among adolescents (Nemati et al., 2020).

This protective effect of social support can be attributed to the emotional, informational, and practical help provided by the support network. This helps adolescents cope more effectively with stress and emotional challenges (Labrague, 2021). In addition, social support fosters a sense of belonging and self-worth in adolescents, which is crucial during the turbulent adolescent years. Therefore, maintaining and strengthening social support networks is critical to promoting adolescents’ mental health and reducing their likelihood of developing NSSI.

### 1.3 Stress-buffering hypothesis and cognitive flexibility

The stress-buffering hypothesis and cognitive flexibility model provide a theoretical framework for understanding the interplay among cognitive flexibility, social support and NSSI behaviors. According to the stress-buffering hypothesis, social support mitigates the negative effects of stress by helping individuals appraise stressful situations as less threatening and providing resources that enhance coping capabilities (Cohen and Wills, 1985). This study also integrates the cognitive flexibility model, a ability to selectively switch between conflicting perspectives and mental processes to appropriately adjust behavior in response to the changing environment (Lan et al., 2023). Social support plays a crucial role in enhancing cognitive flexibility by exposing individuals to diverse perspectives, fostering adaptive problem-solving skills, and creating a supportive environment for exploring alternative solutions (Chen et al., 2021; Calhoun et al., 2022). Enhanced cognitive flexibility, in turn, reduces the likelihood of NSSI by enabling individuals to generate alternative coping strategies and improve problem-solving capabilities (Wolff et al., 2023). Recent research by Lan et al. (2023) further supports this perspective, demonstrating that social support contributes to cognitive reappraisal by offering different ways to interpret a given situation or by helping adolescents develop new explanations for stressful experiences (Lan et al., 2023). This framework suggests that the relationship between social support and reduced NSSI was not merely direct but operates through its enhancement of cognitive flexibility as a mediator.

### 1.4 Gap in our knowledge

Although the roles of cognitive flexibility and social support in influencing adolescent mental health are widely recognized (Uddin, 2021; MacPherson et al., 2022), their interconnections in non-suicidal self-injury are not yet fully understood. Moreover, potential gender differences in these relationships warrant further investigation, as previous research has indicated that the prevalence and correlates of NSSI may vary between male and female adolescents.

By addressing these gaps in our knowledge, this study integrates two theoretical frameworks to explore the relationship between social support, cognitive flexibility, and NSSI among adolescents. The findings may contribute to a more comprehensive understanding of the complex interplay between social support, cognitive processes, and NSSI among Chinese adolescents, ultimately developing targeted interventions to reduce self-injurious behaviors.

## 2 Method

### 2.1 Participants

Participants were recruited across 14 psychiatric hospitals/outpatient clinics or general hospital psychiatric wards of China. Data collection occurred between December 2021 and December 2023, resulting in an initial cohort of 2,109 adolescents. To be eligible for inclusion in the study, participants had to meet specific criteria, which included being aged between 12 and 18 years, having completed a minimum of 6 years of formal education, and providing written informed consent, along with parental or guardian consent. Exclusion criteria encompassed individuals with impairments in reading and writing Chinese characters due to physical or language dysfunction, as well as cases where either the guardian or the individual declined to participate.

### 2.2 Measurements

#### 2.2.1 Demographic information

Demographic information was collected from participants using a self-compiled questionnaire. This questionnaire gathered data on sex, age, and years of education. Sex was recorded as a dichotomous variable coded 0 for male and 1 for female. Age was measured in years since birth. Years of education completed was measured on a continuous scale indicating total years of education starting with first grade.

#### 2.2.2 Social support

Social support was assessed using the 12-item Multidimensional Scale of Perceived Social Support (MSPSS) (Dambi et al., 2018). The MSPSS includes three subscales assessing perceived support from family, friends, and significant others. Participants were asked to rate items on a 7-point Likert scale from 1 (very strongly disagree) to 7 (very strongly agree), with higher scores indicating greater perceived support. In the current study, the Cronbach’s *α* for the total scale was 0.94.

#### 2.2.3 Non-suicidal self-injury

The frequency of non-suicidal self-injury (NSSI) behaviors was assessed using the self-injury section of the Functional Assessment of Self-Mutilation (FASM) (Qu et al., 2021). Participants were presented with 11 items listing various self-harm behaviors such as “cutting,” “biting,” “needle-sticking” and asked how many times they have intentionally engaged in each form of self-harm in the past year. Cronbach’s alpha for FASM in this study was 0.79, indicating acceptable internal consistency.

#### 2.2.4 Cognitive flexibility

Cognitive flexibility was assessed using the Wisconsin Card Sorting Test (WCST) (Miles et al., 2021). The WCST is a neuropsychological test that requires participants to sort cards based on different dimensions (color, shape, number) while receiving feedback on whether each match is correct. It assesses abstract reasoning skills and the ability to shift cognitive strategies based on changing stimulus conditions. Test administration and scoring were performed using the standard procedures outlined in the WCST manual. In the present study, three key indicators from the WCST were utilized: Categories Completed (CC), number of categories successfully completed by the participant; Correct Responses (RC), total number of cards correctly sorted; and Correct Responses Percentage (RCP), the percentage of attempted cards sorted correctly (Fristoe et al., 1997). These indices have been previously used to evaluate WCST performance.

#### 2.2.5 Statistics analysis

Statistical analyses were conducted using SPSS version 26.0 and R version 4.3.2. Descriptive statistics were calculated to characterize the sample. Pearson correlations tested associations between social support, cognitive flexibility, and NSSI. A multiple linear regression analysis examined social support and cognitive performance as predictors of NSSI frequency, controlling for demographics. Standardized betas were reported along with adjust-*R*^2^ and *F* statistics. Mediation model was constructed by structural equation modeling (SEM) in R “lavaan” (Rosseel, 2012). The hypothesized model was tested and model fit indices (chi-square, CFI, NFI, RMSEA) were examined (Kenny et al., 2015). The significance of direct, indirect, and total effects was evaluated using bootstrapping procedures (bootstrap = 5,000). Multi-group analysis was used to determine if path coefficients differed across male and female participants. Measurement invariance was first established by comparing an unconstrained model to a fully constrained model. Structural path values were then compared using critical ratio tests exceeding ±1.96 (Byrne, 2004). The level of significance was set at *p* < 0.05 for all tests.

## 3 Result

### 3.1 Descriptive statistics

The sample consisted of 2,109 adolescents with a mean age of 15.00 years (SD = 1.65; see Table 1 for additional details). Participants completed two cognitive assessments. Correct responses (RC) captured the number of accurate answers out of 100 questions. On average, participants answered 72.75 questions correctly (SD = 17.82). Categories completed (CC) referred to the number of test sections attempted out of 7 total categories. Participants completed 5.04 categories on average (SD = 1.48). The percentage of accurate responses relative to questions attempted (RCP) was also calculated, with participants answering 74% of attempted questions correctly on average (SD = 0.12).

**Table 1:** Descriptive statistics of the study sample.

Variable	*N*	%	*M*	SD
Age			15.00	1.65
Sex				
Male	474	22.48		
Female	1,635	77.52		
Edu years			9.17	1.77
NSSI			19.61	9.76
Social support			46.99	16.75
Correct Responses (RC)			72.75	17.82
Categories Completed (CC)			5.04	1.48
Correct Responses Percentage (RCP)			0.74	0.12

Pearson correlations were conducted between the main study variables (see Table 2). NSSI demonstrated negative correlations with social support (*r* = −0.21, *p* < 0.001) and the cognitive test scores (CC: *r* = −0.12, *p* < 0.001; RCP: *r* = −0.10, *p* < 0.001). This indicates that more frequent NSSI was associated with lower support and worse performance on the cognitive assessments. In contrast, social support showed significant positive correlations with all three cognitive flexibility indexes: RC (*r* = 0.08, *p* < 0.001), CC (*r* = 0.14, *p* < 0.001), and RCP (*r* = 0.12, *p* < 0.001). Therefore, participants reporting more available social support tended to answer more questions correctly, attempt more categories, and have better accuracy. Moreover, the cognitive tests also showed robust interrelationships (*r* > 0.33, *p* < 0.001).

**Table 2:** Correlations between study variables.

	1	2	3	4	5	6	7	8
1. Age	1							
2. Sex	−0.15^***^	1						
3. Edu	0.84^***^	−0.12^***^	1					
4. NSSI	−0.23^***^	0.16^***^	−0.20^***^	1				
5. Social support	0.16^***^	−0.01	0.16^***^	−0.21^***^	1			
6. RC	0.01	−0.01	0.02	−0.02	0.08^***^	1		
7. CC	0.16^***^	−0.04	0.19^***^	−0.12^***^	0.14^***^	0.69^***^	1	
8. RCP	0.10^***^	−0.04	0.13^***^	−0.10^***^	0.12^***^	0.33^***^	0.55^***^	1

NSSI, non-suicidal self-injury; RC, Correct Responses; CC, Categories Completed; RCP, Correct Responses Percentage. ^*^p < 0.05, ^**^p < 0.01, ^***^p < 0.001.

### 3.2 Multiple regression

A multiple linear regression was conducted to predict NSSI frequency using social support, cognitive flexibility index, after controlling for demographic variables (shown in Table 3). The overall model was significant (*F* = 36.06, *p* < 0.001) and explained 11% of the variance in NSSI (Adjusted-*R*^2^ = 0.11). The result of regression revealed that social support (*β* = −0.17, *p* < 0.001), age (*β* = −0.17, *p* < 0.001), and sex (*β* = 0.13, *p* < 0.001) were significantly associated with NSSI. Regarding the cognitive flexibility index, Correct responses was significantly positively predicted NSSI. In contrast, categories completed (*β* = −0.11, *p* = 0.001) significantly negatively predicted NSSI. Correct responses percentage showed a non-significant negative trend (*β* = −0.10, *p* > 0.05).

**Table 3:** Regression results predicting NSSI.

Model		*β*	*SE*	*t*	*p*	VIF
Independent variable	Social support	−0.17	0.02	8.00	<0.001	1.04
	Cognitive flexibility					
	RC	0.08	0.03	2.76	0.006	1.94
	CC	−0.11	0.03	3.20	0.001	2.53
	RCP	−0.03	0.02	1.05	0.30	1.44
Control variables	Age	−0.17	0.02	7.68	< 0.001	1.10
	Edu	0.03	0.02	1.21	0.23	1.01
	Sex	0.13	0.02	6.13	< 0.001	1.02
*F*	36.06^***^					
Adjusted-*R*^2^	0.11					

^***^p < 0.001.

### 3.3 Mediation analysis

**Figure 1:**
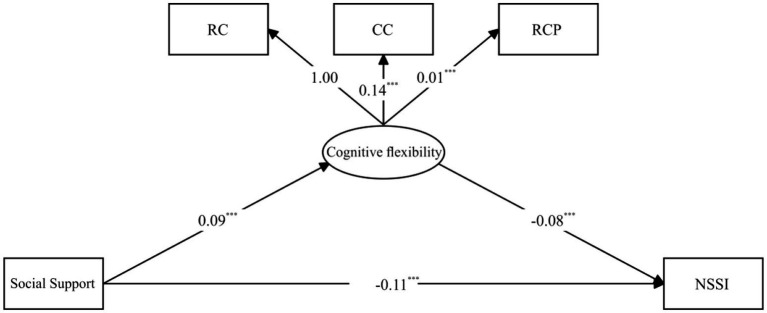
Mediation model path visualization. NSSI, non-suicidal self-injury; RC, Correct Responses; CC, Categories Completed; RCP, Correct Responses Percentage. ^*^p < 0.05, ^**^p < 0.01, ^***^p < 0.001.

While multiple regression analysis allowed to examine the predictive effect of each factor on NSSI separately, cognitive flexibility is a multidimensional construct consisting of several related but distinct indicators. Therefore, in the current study, SEM was used to model cognitive flexibility as a latent variable. SEM assessed the mediatory role of cognitive flexibility in the social support-NSSI relationship, as shown in Figure 1. The proposed model exhibited great fit (*χ*^2^ = 22.52, CFI = 0.99, NFI = 0.99, RMSEA = 0.047). In this model, social support was a significant positive predictor of cognitive flexibility (*β* = 0.09 *p* < 0.001). Subsequently, cognitive flexibility significantly predicted NSSI (*β* = −0.08, *p* < 0.001). Additionally, social support directly influenced NSSI (*β* = 0.11, *p* < 0.001) even after accounting for the indirect pathway via cognitive flexibility. Importantly, the mediation effect was statistically significant [*β* = −0.01 95% CI (−0.01, −0.01)], explaining approximately 8.33% of social support‘s total effect on NSSI. Table 4 elaborates on these effects.

**Table 4:** Effect decomposition for the mediation model.

	Effect	95% CI	*Z*	*p*
Direct	−0.11	[−0.13, -0.09]	9.14	< 0.001
Indirect	−0.01	[−0.01, −0.01]	3.73	< 0.001
Total direct	−0.12	[−0.14, −0.10]	9.72	< 0.001

### 3.4 Sex variance of the structural model

To test measurement variance across sexes, a multi-group analysis was conducted comparing an unconstrained model to a model with structural path coefficients constrained to equality. The unconstrained model showed acceptable fit (*χ*^2^ = 20.49, CFI = 0.99, NFI = 0.99, RMSEA = 0.04). The constrained model also demonstrated good fit (*χ*^2^ = 34.63, CFI = 0.99, NFI = 0.99, RMSEA = 0.05). The chi-square test revealed the constrained model was significantly worse fitting compared to the unconstrained model (Δ*χ*^2^ = 14.15, *p* = 0.003). Critical ratio comparisons revealed a significant sex difference for the path from social support to cognitive performance. Specifically, the linkage was stronger among females (*β* = 0.11, *p* < 0.001) compared to males (*β* = 0.02, *p* > 0.05), with the ratio of 5.50 exceeding 1.96 (refer to Table 5).

**Table 5:** Multi-group critical ratio analysis.

Path coefficient	Male	Female	Critical ratios
Social support → Cognitive flexibility	0.02	0.11^***^	5.50^**^
Cognitive flexibility → NSSI	−0.07^*^	−0.08^***^	1.14
Social support → NSSI	−0.08^**^	−0.12^***^	1.50

^*^p < 0.05, ^**^p < 0.01, ^***^p < 0.001.

## 4 Discussion

The core hypothesis of our study aimed to reveal the complex interplay between social support, cognitive flexibility, and NSSI among Chinese adolescents. This study makes an important contribution to a deeper understanding of non-suicidal self-injurious behavior among Chinese adolescents. Notably, our findings are consistent with recent literature that suggests perceived social support is negatively associated with the frequency of nonsuicidal self-injury, emphasizing the protective role of social support in reducing nonsuicidal self-injurious behavior (Nemati et al., 2020). Similarly, our findings are consistent with the findings that there is a link between cognitive flexibility and nonsuicidal self-injury. Specifically, higher levels of cognitive flexibility are associated with a lower propensity for NSSI behaviors in adolescents (Morea and Calvete, 2021). Furthermore, our analyses confirm existing research that significant gender differences exist in the dynamics of social support, cognitive functioning, and NSSI (Wan et al., 2019; Kellerman et al., 2021). A noteworthy phenomenon is the stronger correlation between social support and cognitive functioning in female adolescents, suggesting gender differences in the relationship between these variables and the NSSI.

### 4.1 Social support: a multidimensional buffer against NSSI

Our study found a significant negative association between social support and the frequency of non-suicidal self-injury (NSSI) in adolescents. This is consistent with research highlighting the protective effects of strong social networks on adolescent mental health. For example, social support was found to mediate the relationship between anxiety-mediated negative life events and NSSI among Chinese adolescents, suggesting the importance of social support in mitigating self-injurious behaviors (Zhang et al., 2022). Furthermore, another study reported that family support was negatively associated with the frequency of NSSI behaviors among Chinese adolescents with mood disorders, emphasizing the value of family relationships (Meng et al., 2022). Furthermore, recent research has highlighted that the absence of a supportive environment can significantly increase vulnerability to NSSI behaviors (Iswanti et al., 2024).

Our study also adds a cultural dimension to the understanding of NSSI by extending prior knowledge by quantifying this relationship in a specific group of Chinese adolescents. This is crucial given the cultural differences in family factors and social structures in China. Nemati et al. (2020) highlighted that weak family psychological functioning and low levels of perceived social support significantly increase the odds of adolescents experiencing NSSI, suggesting the influence of culture on these dynamics (Mackin et al., 2017; Wan et al., 2019). The specific cultural context of China may influence the perception and impact of social support in different ways compared to Western contexts, so our research insights are particularly relevant to this population.

### 4.2 The mediating role of cognitive flexibility

This study reveals a significant mediating role of cognitive flexibility in the relationship between social support and non-suicidal self-injury (NSSI) among adolescents. This mediation effect, accounting for 8.33% of the total effect of social support on NSSI, suggests that social support may partially exert its protective influence by enhancing cognitive flexibility. Social support appears to foster diverse perspectives and problem-solving strategies, thereby enhancing an adolescents ability to think flexibly (Zhang et al., 2022). This enhanced cognitive flexibility, in turn, enables adolescents to generate alternative solutions to distress, reducing the likelihood of resorting to NSSI (Polanco-Roman et al., 2015). The inverse relationship between cognitive flexibility and NSSI aligns with previous research showing that deficits in problem-solving skills and lower psychological flexibility are associated with NSSI behaviors (Park and Ammerman, 2023). Furthermore, cognitive flexibility serves as a resilience factor, enabling adolescents to employ adaptive problem-solving strategies in stressful situations (Shen et al., 2024). This partial mediation indicates that while cognitive flexibility plays a significant role, other factors also contribute to the protective effect of social support against NSSI. The findings extend beyond the traditional focus on impulsivity in NSSI research, offering a more nuanced and proactive approach to understanding and preventing NSSI. By highlighting the interconnectedness of cognitive flexibility with other developmental abilities, such as emotion regulation and social skills, the study provides valuable insights for developing targeted interventions to mitigate NSSI risk among adolescents.

### 4.3 Sex differences in the impact of social support and cognitive flexibility

Our study shows that the association between social support and cognitive performance is stronger in women than in men, providing novel and important insights into gender differences in adolescent mental health. This finding is not only consistent with previous research showing that women have higher rates of non-suicidal self-injury (NSSI), but more in-depth research suggests that underlying cognitive and social factors play a role in women’s non-suicidal self-injury (NSSI) (Wan et al., 2019). Play an important role in suicidal self-injury (NSSI) role in this difference.

The reasons behind these gender differences are likely multifactorial. There are often significant differences in the social expectations of males and females (Grossman and Wood, 1993), which may influence how adolescents perceive and receive social support. For example, women may be more encouraged to seek and value emotional support, which may enhance their cognitive abilities to cope with stressors such as NSSI. Conversely, social norms may prevent men from expressing vulnerability, potentially affecting their ability to benefit from existing social supports.

Differences in emotional expression and processing between the sexes provide another layer of explanation. Women typically become more expressive and emotionally regulated during socialization, which can promote better emotional regulation through social support networks (Shangguan et al., 2022). This emotional attunement can enhance cognitive flexibility and help adapt to challenging situations without resorting to harmful behaviors such as NSSI. Conversely, if men are less willing or able to process emotions socially, this may impact the efficacy of social support on their cognitive performance.

### 4.4 Limitations and future direction

Although our study provides insights into the interplay between social support, cognitive flexibility, and NSSI in Chinese adolescents, there are still certain limitations. First, the sample was drawn from outpatient and inpatient facilities across provinces and may not be representative of the broader adolescent population (Omair, 2014). This limits the generalizability of our findings, particularly to adolescents not engaged in mental health services. Although self-report measures are validated, their use may introduce biases such as social desirability or inaccurate recall (Giromini et al., 2022). Cross-sectional designs limit our ability to infer causal relationships between the variables studied, thereby limiting the directionality of understanding these relationships.

Future research should consider longitudinal designs to establish causal relationships and track the progression of NSSI behaviors over time (Guo et al., 2024). Diversifying the sample to include nonclinical populations will enhance the generalizability of the study findings. Intervention studies focused on enhancing social support and cognitive flexibility may provide practical insights into effective NSSI prevention strategies. Furthermore, exploring these relationships in different cultural contexts will broaden the mechanisms underlying NSSI. Employing mixed methods, combining self-reports with objective assessments, can mitigate the bias inherent in self-report measures and provide a more nuanced view of these complex phenomena.

## 5 Conclusion

Our study significantly advances the understanding of NSSI among Chinese adolescents by exploring the interplay between social support, cognitive flexibility, and NSSI. These findings highlight the protective role of social support and the importance of cognitive flexibility, providing important insights for the development of targeted interventions to mitigate NSSI risk. Also, a comprehensive, culturally sensitive approach to adolescent mental health is emphasized to guide future research and mental health practice.

## Data Availability

The raw data supporting the conclusions of this article will be made available by the authors, without undue reservation.

## References

[ref1] Bozorgi Kazerooni A. Gholamipour N. (2023). Investigating the moderating role of cognitive flexibility in the relationship between maltreatment and emotion regulation in adolescence with childhood trauma. J. Res. Health 13, 133–142. doi: 10.32598/JRH.13.2.2075.1

[ref2] Brunner R. Kaess M. Parzer P. Fischer G. Resch F. Carli V.. (2013). 3038–characteristics of non-suicidal self-injury and suicide attempts among adolescents in Europe: results from the European research consortium seyle. Eur. Psychiatry 28:1. doi: 10.1016/S0924-9338(13)77531-X, PMID: 21920709

[ref3] Byrne B. M. (2004). Testing for multigroup invariance using AMOS graphics: a road less traveled. Struct. Equ. Model. Multidiscip. J. 11, 272–300. doi: 10.1207/s15328007sem1102_8

[ref4] Calhoun C. D. Stone K. J. Cobb A. R. Patterson M. W. Danielson C. K. Bendezú J. J. (2022). The role of social support in coping with psychological trauma: an integrated biopsychosocial model for posttraumatic stress recovery. Psychiatry Q. 93, 949–970. doi: 10.1007/s11126-022-10003-w, PMID: 36199000 PMC9534006

[ref5] Chen H. Hong L. Tong S. Li M. Sun S. Xu Y.. (2023). Cognitive impairment and factors influencing depression in adolescents with suicidal and self-injury behaviors: a cross-sectional study. BMC Psychiatry 23:247. doi: 10.1186/s12888-023-04726-8, PMID: 37046299 PMC10099683

[ref6] Chen L. Zilioli S. Jiang Y. Wang X. Lin D. (2021). Perceived social support and children’s physiological responses to stress: an examination of the stress-buffering hypothesis. Psychosom. Med. 83, 51–61. doi: 10.1097/PSY.0000000000000875, PMID: 33060454

[ref7] Ciranka S. van den Bos W. (2021). Adolescent risk-taking in the context of exploration and social influence. Dev. Rev. 61:100979. doi: 10.1016/j.dr.2021.100979

[ref8] Cohen S. Wills T. A. (1985). Stress, social support, and the buffering hypothesis. Psychol. Bull. 98, 310–357. doi: 10.1037/0033-2909.98.2.310 3901065

[ref9] Dambi J. M. Corten L. Chiwaridzo M. Jack H. Mlambo T. Jelsma J. (2018). A systematic review of the psychometric properties of the cross-cultural translations and adaptations of the multidimensional perceived social support scale (MSPSS). Health Qual. Life Outcomes 16:80. doi: 10.1186/s12955-018-0912-0, PMID: 29716589 PMC5930820

[ref10] Fan Y. Liu J. Zeng Y. Conrad R. Tang Y. (2021). Factors associated with non-suicidal self-injury in Chinese adolescents: a meta-analysis. Front. Psych. 12:747031. doi: 10.3389/fpsyt.2021.747031, PMID: 34916971 PMC8669619

[ref11] Fristoe N. M. Salthouse T. A. Woodard J. L. (1997). Examination of age-related deficits on the Wisconsin card sorting test. Neuropsychology 11, 428–436. doi: 10.1037/0894-4105.11.3.428 9223147

[ref12] Giromini L. Young G. Sellbom M. (2022). Assessing negative response bias using self-report measures: new articles, new issues. Psychol. Inj. Law 15, 1–21. doi: 10.1007/s12207-022-09444-2

[ref13] Grossman M. Wood W. (1993). Sex differences in intensity of emotional experience: a social role interpretation. J. Pers. Soc. Psychol. 65, 1010–1022. doi: 10.1037/0022-3514.65.5.1010, PMID: 8246109

[ref14] Guo X. Wang L. Li Z. Feng Z. Lu L. Jiang L.. (2024). Factors and pathways of non-suicidal self-injury in children: insights from computational causal analysis. Front. Public Health 12:1305746. doi: 10.3389/fpubh.2024.1305746, PMID: 38532971 PMC10963487

[ref15] Heffner J. Son J.-Y. FeldmanHall O. (2021). Emotion prediction errors guide socially adaptive behaviour. Nat. Hum. Behav. 5, 1391–1401. doi: 10.1038/s41562-021-01213-6, PMID: 34667302 PMC8544818

[ref16] Iswanti D. I. Lai L.-L. Saifudin I. M. M. Y. Kandar K. Dewi R. K. Cahyaningrum D. D. (2024). The predictor of non-suicidal self-injury behavior among adolescents: a cross-sectional study. J. Ners 19, 125–133. doi: 10.20473/jn.v19i2.54610

[ref17] Kellerman J. Millner A. Joyce V. W. Nash C. C. Buonopane R. J. Nock M.. (2021). Social support and nonsuicidal self-injury among adolescent psychiatric inpatients. Res. Child Adolesc. Psychopathol. 50, 1351–1361. doi: 10.1007/s10802-022-00931-3, PMID: 35579780 PMC10773970

[ref18] Kenny D. A. Kaniskan B. McCoach D. B. (2015). The performance of RMSEA in models with small degrees of freedom. Sociol. Methods Res. 44, 486–507. doi: 10.1177/0049124114543236

[ref19] Labrague L. J. (2021). Psychological resilience, coping behaviours and social support among health care workers during the COVID-19 pandemic: a systematic review of quantitative studies. J. Nurs. Manag. 29, 1893–1905. doi: 10.1111/jonm.13336, PMID: 33843087 PMC8250179

[ref20] Lan X. Ma C. Ma Y. (2023). “Three pills” (cognitive reappraisal × social support × cognitive flexibility) and their impact on ADHD symptoms in early adolescence: synergistic or compensatory effect? Personal. Individ. Differ. 211:112246. doi: 10.1016/j.paid.2023.112246

[ref21] Lim K.-S. Wong C. H. McIntyre R. S. Wang J. Zhang Z. Tran B. X.. (2019). Global lifetime and 12-month prevalence of suicidal behavior, deliberate self-harm and non-suicidal self-injury in children and adolescents between 1989 and 2018: a meta-analysis. Int. J. Environ. Res. Public Health 16:4581. doi: 10.3390/ijerph16224581, PMID: 31752375 PMC6888476

[ref22] Mackin D. M. Perlman G. Davila J. Kotov R. Klein D. N. (2017). Social support buffers the effect of interpersonal life stress on suicidal ideation and self-injury during adolescence. Psychol. Med. 47, 1149–1161. doi: 10.1017/S0033291716003275, PMID: 27995812

[ref23] MacPherson H. A. Kudinova A. Y. Schettini E. Jenkins G. A. Gilbert A. C. Thomas S. A.. (2022). Relationship between cognitive flexibility and subsequent course of mood symptoms and suicidal ideation in young adults with childhood-onset bipolar disorder. Eur. Child Adolesc. Psychiatry 31, 299–312. doi: 10.1007/s00787-020-01688-0, PMID: 33392723 PMC8253874

[ref24] Martín-Rodríguez A. Tornero-Aguilera J. F. López-Pérez P. J. Clemente-Suárez V. J. (2022). Overweight and executive functions, psychological and behavioral profile of Spanish adolescents. Physiol. Behav. 254:113901. doi: 10.1016/j.physbeh.2022.113901, PMID: 35810837

[ref25] Meng L. Qu D. Bu H. Huo L. Qi L. Yang J.. (2022). The psychosocial correlates of non-suicidal self-injury within a sample of adolescents with mood disorder. Front. Public Health 10:768400. doi: 10.3389/fpubh.2022.768400, PMID: 35273935 PMC8902037

[ref26] Miles S. Howlett C. A. Berryman C. Nedeljkovic M. Moseley G. L. Phillipou A. (2021). Considerations for using the Wisconsin card sorting test to assess cognitive flexibility. Behav. Res. Methods 53, 2083–2091. doi: 10.3758/s13428-021-01551-3, PMID: 33754321

[ref27] Morea A. Calvete E. (2021). Cognitive flexibility and selective Attention’s associations with internalizing symptoms in adolescents: are they reciprocal? J. Youth Adolesc. 50, 921–934. doi: 10.1007/s10964-021-01402-6, PMID: 33575916

[ref28] Nemati H. Sahebihagh M. H. Mahmoodi M. Ghiasi A. Ebrahimi H. Atri S. B.. (2020). Non-suicidal self-injury and its relationship with family psychological function and perceived social support among Iranian high school students. J. Res. Health Sci. 20:e00469. doi: 10.34172/jrhs.2020.04, PMID: 32814690 PMC7585761

[ref29] Omair A. (2014). Sample size estimation and sampling techniques for selecting a representative sample. J. Health Spec. 2:142. doi: 10.4103/1658-600X.142783

[ref30] Park Y. Ammerman B. A. (2023). For better or worse?: The role of cognitive flexibility in the association between nonsuicidal self-injury and suicide attempt. J. Psychiatr. Res. 158, 157–164. doi: 10.1016/j.jpsychires.2022.12.040 36586214

[ref31] Polanco-Roman L. Jurska J. Quiñones V. Miranda R. (2015). Brooding, reflection, and distraction: relation to non-suicidal self-injury versus suicide attempts. Arch. Suicide Res. 19, 350–365. doi: 10.1080/13811118.2014.981623, PMID: 25517765 PMC4867417

[ref32] Qu D. Wang Y. Zhang Z. Meng L. Zhu F. Zheng T.. (2021). Psychometric properties of the Chinese version of the functional assessment of self-mutilation (FASM) in Chinese clinical adolescents. Front. Psych. 12:755857. doi: 10.3389/fpsyt.2021.755857, PMID: 35153848 PMC8826685

[ref33] Rosseel Y. (2012). Lavaan: an R package for structural equation modeling. J. Stat. Softw. 48, 1–36. doi: 10.18637/jss.v048.i02

[ref34] Shangguan C. Zhang L. Wang Y. Wang W. Shan M. Liu F. (2022). Expressive flexibility and mental health: the mediating role of social support and gender differences. Int. J. Environ. Res. Public Health 19:456. doi: 10.3390/ijerph19010456, PMID: 35010716 PMC8744810

[ref35] Shen G. Li C.-H. Ruan Q.-N. Xu S. Yan W.-J. (2024). Assessing the contributions of gender, clinical symptoms, and psychometric traits to non-suicidal self-injury behaviors in Chinese adolescents: a nomogram approach. Child Adolesc. Psychiatry Ment. Health 18:139. doi: 10.1186/s13034-024-00832-x, PMID: 39501322 PMC11536789

[ref36] Uddin L. Q. (2021). Cognitive and behavioural flexibility: neural mechanisms and clinical considerations. Nat. Rev. Neurosci. 22, 167–179. doi: 10.1038/s41583-021-00428-w, PMID: 33536614 PMC7856857

[ref37] Wan Y. Chen R. Ma S. McFeeters D. Sun Y. Hao J.. (2019). Associations of adverse childhood experiences and social support with self-injurious behaviour and suicidality in adolescents. Br. J. Psychiatry 214, 146–152. doi: 10.1192/bjp.2018.263, PMID: 30477603 PMC6429251

[ref38] Wang Y. Chung M. C. Wang N. Yu X. Kenardy J. (2021). Social support and posttraumatic stress disorder: a meta-analysis of longitudinal studies. Clin. Psychol. Rev. 85:101998. doi: 10.1016/j.cpr.2021.101998, PMID: 33714168

[ref39] Washburn J. J. Richardt S. L. Styer D. M. Gebhardt M. Juzwin K. R. Yourek A.. (2012). Psychotherapeutic approaches to non-suicidal self-injury in adolescents. Child Adolesc. Psychiatry Ment. Health 6:14. doi: 10.1186/1753-2000-6-14, PMID: 22463499 PMC3782878

[ref40] Wolff B. Franco V. R. Magiati I. Pestell C. F. Glasson E. J. (2023). Psychosocial and neurocognitive correlates of suicidal thoughts and behaviours amongst siblings of persons with and without neurodevelopmental conditions. Res. Dev. Disabil. 139:104566. doi: 10.1016/j.ridd.2023.104566, PMID: 37441861

[ref41] Zhang Y. Suo X.-S. Zhang Y. Zhang S. Yang M. Qian L.. (2022). The relationship between negative life events and nonsuicidal self-injury among Chinese adolescents: a moderated-mediation model. Neuropsychiatr. Dis. Treat. 18, 2881–2890. doi: 10.2147/NDT.S386179, PMID: 36540672 PMC9760044

[ref42] Zhou Q. Liang Y. Gao Y. Liu X. (2024). Social support and non-suicidal self-injury in adolescents: the differential influences of family, friends, and teachers. J. Youth Adolesc. 54, 414–425. doi: 10.1007/s10964-024-02066-8, PMID: 39127815

